# Next-generation TREM2-targeted therapies for Alzheimer's disease: insights and directions from the INVOKE-2 trial

**DOI:** 10.3389/fnagi.2026.1837720

**Published:** 2026-06-11

**Authors:** Mingsong Shi

**Affiliations:** 1 National Health Commission Key Laboratory of Nuclear Technology Medical Transformation, Mianyang Central Hospital, School of Medicine, University of Electronic Science and Technology of China, Mianyang, Sichuan, China; 2 Sichuan Provincial Engineering Research Center of Nuclear Medical Equipment Translation and Application, Mianyang Central Hospital, School of Medicine, University of Electronic Science and Technology of China, Mianyang, Sichuan, China

**Keywords:** agonistic, Alzheimer's disease, clinical trial, microglia, precision medicine, triggering receptor expressed on myeloid cells 2

## 1 Introduction

Alzheimer's disease (AD) is a progressive neurodegenerative disorder that leads to irreversible cognitive decline, memory loss, and the erosion of personal autonomy, placing immense burden on patients, families, and healthcare systems worldwide. By 2050, the global population of patients with AD is estimated to exceed 139 million (Clare et al., 2025). Therefore, the development of effective disease-modifying therapies for AD is urgently required.

Current therapeutic strategies have focused on disease-modifying and symptomatic treatments (Zhang et al., 2024). For example, drugs such as Lecanemab, Aducanumab, and Bepranemab directly target the amyloid-beta (Aβ) plaques and tau neurofibrillary tangles, which are classical histopathologies of AD. Although some anti-amyloid monoclonal antibodies, such as donanemab and lecanemab (Sims et al., 2023; Van Dyck et al., 2023), are milestones for AD treatment. The side effects, such as amyloid-related imaging abnormalities [ARIA; i.e., vasogenic edema/effusion (ARIA-E) and microhemorrhages/hemosiderin deposits (ARIA-H)], have been shown in clinical application (Cummings, 2025). Therefore, novel treatment strategies must be developed for AD.

TREM2-mediated neuroinflammation is advantageous in early AD stages (e.g., enhancing Aβ clearance), but this effect is strictly stage-specific. AD treatment strategies involve the removal of pathological proteins to modulate the immune microenvironment. Triggering receptor expressed on myeloid cell 2 (TREM2), a key regulator of microglial function, is one of the main targets for modulating the immune microenvironment (Qi et al., 2025). Human genetic studies have shown that TREM2 variants (such as R47H, a loss-of-function variant) are associated with two- to four-fold increase in the risk of late-onset AD (Guerreiro et al., 2013; Jonsson et al., 2013). TREM2 enables microglia to sense pathological insults, clear Aβ debris, and regulate inflammatory responses (Cheng-Hathaway et al., 2018; Mallika et al., 2026). Therefore, the pharmacological enhancement of TREM2 activity represents a rational strategy for modifying AD progression by controlling microglial protective functions.

A recent publication of the INVOKE-2 trial in *Nature Medicine* provided critical results for the treatment of AD in humans by targeting TREM2 (Mayorga et al., 2025; Mummery et al., 2026). This current *Opinion Article* discusses the unresolved complexities of TREM2-targeting drugs based on the findings from INVOIKE-2 and future research on TREM2 drug development.

## 2 Intervention timing challenges in early AD

The INVOKE-2 trial evaluated the TREM2 agonist antibody AL002 in early AD (Mummery et al., 2026). Although the study did not meet its primary endpoint of slowing cognitive decline, it provided critical human data on TREM2-targeted therapy. AL002 reduced soluble TREM2 and increased osteopontin levels in cerebrospinal fluid, confirming target engagement. However, this did not translate into clinical benefits. Although the INVOKE-2 cohort was strictly characterized by early-stage AD (e.g., excluding APOE ε4/ε4 carriers and featuring moderate baseline p-tau217 levels), there remains a notable heterogeneity in the intrinsic stage of neuroinflammation. Consequently, a subset of patients may have already transitioned past the optimal therapeutic window where TREM2 agonism effectively enhances Aβ clearance. Thus, these results suggest that intervention timing, such as the initiation of TREM2 activation, must be carefully determined for the treatment of AD by targeting TREM2.

Preclinical and Phase I clinical results support the dynamic stage-dependent role of TREM2 in AD pathogenesis (Long et al., 2024). TREM2-mediated microglial activation enhances Aβ clearance and exerts neuroprotective effects in early disease phases (Qi et al., 2025). However, chronic or excessive activation may exacerbate neuroinflammation and tau pathology in the later stages (Jain et al., 2022). TREM2 agonism reduces the amyloid burden and improves cognition in the early disease course, whereas delayed or sustained activation of TREM2 can lead to increased axonal dystrophy and synaptic loss in AD pathology. This biphasic role of TREM2 emphasizes that the therapeutic efficacy of TREM2 agonism depends not only on target engagement, but also on the intervention time with disease biology.

From the INVOKE-2 trial, future clinical strategies should focus on defining and targeting the precise therapeutic window for TREM2 regulation. Biomarkers related to distinct AD stages need to be developed and validated, such as soluble TREM2 (sTREM2), neuroinflammatory markers, and tau positron emission tomography in cerebrospinal fluid or plasma (Colonna and Holtzman, 2025). These biomarkers can be used to identify patients in early inflammation-modifiable phases of AD. Clinical trial designs should incorporate adaptive enrichment strategies to select participants based on their microglial activity profiles or early pathological burdens. Additionally, the optimal time points for combination therapy with anti-amyloid or anti-tau agents should be investigated to determine whether synergistic effects can enhance clinical outcomes. Ultimately, a personalized, biomarker-guided framework is essential for identifying the potential therapeutic window of TREM2-targeted therapies for AD.

## 3 Complex TREM2 biology and targeting drug activity

The INVOKE-2 trial results demonstrated the biological complexity of TREM2 in AD. AL002 successfully engaged its target, as evidenced by reduced sTREM2 levels in the cerebrospinal fluid. However, this pharmacodynamic effect did not translate into clinical efficacy. This highlights the potential contradiction between activating signaling and reducing sTREM2 expression. Although AL002 activates signaling to promote full-length TREM2 internalization and degradation, sTREM2 reduction may inadvertently weaken the beneficial functions of sTREM2. Thus, simple receptor activation of TREM2 may not be sufficient, and a better understanding of TREM2 biology is required for effective drug design.

Future drug development that targets TREM2 must extend beyond broad receptor agonists such as AL002. Strategies should aim to achieve high selectivity and precision. One approach specifically amplifies endogenous TREM2 signaling at the membrane without affecting ectodomain shedding. For example, small molecules bind to the juxtamembrane region and allosteric site of TREM2 (such as VG-3927, C1, MTX46943, and EN020) (Mirescu, 2025; Polydoro et al., 2025; Yuan et al., 2025; Cho et al., 2026). Another method differentially modulates the distinct functions of full-length TREM2 and sTREM2, as exemplified by antibody antagonists such as VHB937, VGL101, and DNL919 (Van Lengerich et al., 2023; Feuerbach et al., 2025; Meier et al., 2025; Zhang et al., 2026). Furthermore, drugs need to specifically enhance beneficial microglial activities—such as Aβ phagocytosis and plaque compaction—without inducing widespread pro-inflammatory responses. Ultimately, dissecting the complex signaling networks downstream of TREM2 and sTREM2 will be critical for developing the next generation of effective and safe immunomodulatory therapies for AD.

## 4 Safety beyond amyloid clearance from the TREM2 agonist AL002

The INVOKE-2 trial demonstrated that treatment with the TREM2 agonist AL002 was associated with an increased incidence of ARIA. This did not decrease with the brain amyloid burden. The risk of ARIA, including edema (ARIA-E) and microhemorrhages (ARIA-H), is strongly associated with the APOE ε4 genotype (Mummery et al., 2026). Fifteen APOE ε4/ε4 participants were initially enrolled in Part 1 of the INVOKE-2 trial, but their participation was discontinued early due to a significantly higher incidence and severity of ARIA; subsequent enrollment of this genotype was permanently halted, and these cases were excluded from the full analysis set. This indicates that TREM2 activation can trigger vascular inflammatory responses and cerebrovascular injury through mechanisms independent of amyloid plaque clearance.

TREM2-positive perivascular macrophages are involved in vascular inflammation (Taylor et al., 2024; Wang et al., 2026). Activation of these cells by anti-amyloid immunotherapy or TREM2 agonism can lead to microhemorrhages and vasogenic edema. The APOE ε4 genotype is a known risk factor for cerebral amyloid angiopathy and severe ARIA. This suggests that a signaling pathway for immune activation around amyloid-laden vessels disrupts vascular integrity, particularly in APOE ε4 carriers. The biology of APOE ε4 carriers involves a drug-related inflammatory response rather than rapid parenchymal amyloid clearance.

Future research must preferentially understand and mitigate these safety risks, such as the specific role of TREM2 signaling in perivascular macrophages and other brain barrier-associated immune cells. The molecular relationship between the APOE ε4 genotype and exacerbated vascular inflammation upon TREM2 activation also needs to be elucidated. Drug development strategies should include designing next-generation TREM2 modulators to enhance microglial activation and reduce harmful perivascular inflammation. Furthermore, clinical trials must incorporate rigorous safety monitoring, especially in APOE ε4 homozygote carriers. A detailed disease classification based on vascular amyloid burden and genotype must be considered to improve the management of ARIA risk.

## 5 Precision approaches for future TREM2-targeting drug development

The INVOKE-2 trial highlighted the key role of precision medicine in TREM2-targeted therapies. The trial results highlighted three critical challenges in drug development. First, the therapeutic effects of TREM2 agonistic antibodies are highly dependent on the stage of AD. Secondly, the current TREM2 agonist approach lacks sufficient selectivity for beneficial signaling pathways. Third, patient heterogeneity, particularly genetics and baseline neuroinflammation, significantly influences treatment response and safety. This indicates that a simple single approach may be inadequate for the identification of complex immunomodulatory targets.

TREM2 function and microglial states change dynamically across the AD continuum (Gratuze et al., 2018). Structural biology studies have shown that the protein–protein interaction interface for TREM2 binding with biological substrates can be applied as a drug-binding pocket (Qi et al., 2025). Furthermore, both TREM2 variants (e.g., R47H) and APOE genotypes alter disease risk and microglial function. Biomarkers such as soluble TREM2, GFAP, and YKL-40 in cerebrospinal fluid or plasma have emerged as indicators of microglial activation and treatment engagement (Benedet et al., 2021). These results explore the basis for a precise therapeutic strategy.

Future studies should integrate multi-dimensional precision approaches. First, the optimal therapeutic window should be defined using stage-specific biomarkers to enroll patients in the early or preclinical phases. Second, next-generation TREM2 modulators should be applied with pharmacological precision, such as blood–brain barrier small molecules or engineered antibodies, which can balance specific TREM2 signaling branches and soluble TREM2 function. Third, a precise predictive model for patients with AD is necessary for TREM2 agonist treatment. For example, genetic profiling (TREM2/APOE genotype), advanced neuroimaging (to assess vascular amyloid and inflammation), and fluid biomarkers have been combined to identify responders and stratify them according to ARIA risk (Salloway et al., 2025; Wang et al., 2026). Application of this integrated strategy is crucial for enhancing the TREM2 agonistic therapeutic potential for AD.
